# Incidence of Type 2 Diabetes Mellitus in Transgender Individuals Undergoing Gender Affirming Hormonal Therapy: A Systematic Review

**DOI:** 10.7759/cureus.58137

**Published:** 2024-04-12

**Authors:** Priyanka Panday, Samrah Ejaz, Simhachalam Gurugubelli, Suviksh K Prathi, Yaneisi Palou Martinez, Divine Besong Arrey Agbor, Tuheen Sankar Nath

**Affiliations:** 1 Research, California Institute of Behavioral Neurosciences & Psychology, Fairfield, USA; 2 Internal Medicine, Meharry Medical College, Nashville, USA; 3 Internal Medicine, California Institute of Behavioral Neurosciences & Psychology, Fairfield, USA; 4 Internal Medicine, Memorial Healthcare, Gulfport, USA; 5 Medicine, California Institute of Behavioral Neurosciences & Psychology, Fairfield, USA; 6 Medicine, St. George's University School of Medicine, St. Georges, GRD; 7 Clinical Research and Internal Medicine, California Institute of Behavioral Neurosciences & Psychology, Fairfield, USA; 8 Internal Medicine, Richmond University Medical Center, Staten Island, USA; 9 Surgical Oncology, California Institute of Behavioral Neurosciences & Psychology, Fairfield, USA; 10 Surgical Oncology, Tata Medical Centre, Kolkata, IND

**Keywords:** transmen, transwomen, diabetes mellitus type 2, gender dysphoria, testosterone, estrogen, insulin resistance, diabetes, transgender, gaht

## Abstract

Gender-affirming hormonal therapy (GAHT), which includes estrogen, testosterone, androgen agonists, is commonly used in transgender individuals to change their secondary sexual characteristics to align with their gender identity. However, this treatment could result in metabolic side effects that could increase the chances of acquiring type 2 diabetes mellitus. Thus, this study aims to compare differences in body mass index (BMI), insulin resistance, and the incidence of type 2 diabetes mellitus between cisgender and transgender individuals undergoing GAHT. Following Preferred Reporting Items for Systematic Reviews and Meta-Analyses (PRISMA) standards, we conducted a systematic review searching through PubMed, Google Scholar, Medline (Medical Literature Analysis and Retrieval System Online), and ResearchGate for articles published between 2014 and 2024. The final search was conducted in February 2024. Out of the 3,934 articles reviewed, 11 were selected, focusing on insulin sensitivity/resistance, diabetes incidence, and BMI changes with GAHT. Although our result findings did not show clear evidence of increased diabetes incidence among GAHT patients, it was observed that GAHT does increase BMI and insulin resistance in transgender individuals. Notably, compared to transgender men, transgender women on GAHT were found to be more prone to insulin resistance. We recommend regularly monitoring insulin sensitivity parameters and HbA1c during GAHT to monitor metabolic side effects. Further research and more clinical trials are needed to confirm the GAHT's impact on insulin resistance and to evaluate its role in the onset of type 2 diabetes mellitus.

## Introduction and background

As per the International Classification of Diseases 11th Revision (ICD-11), gender incongruence is a sexual health condition distinguished by a significant and enduring misalignment between an individual’s perceived gender and their assigned sex [1]. Transgender women identify as female despite being assigned as male at birth, while transgender men identify as male despite being assigned as female at birth (ICD-11) [1]. The anxiety that people may feel because of the mismatch between their perceived gender identity and the sex they were assigned at birth is known as gender dysphoria. The primary objective of therapy is to mitigate this anxiety and promote psychological well-being [2]. The precise prevalence of transgender individuals and gender dysphoria remains not precisely known and is likely underestimated due to several factors including ambiguous terminology [3]. Population-based studies have indicated a higher prevalence than clinical estimates, ranging from 0.5% in the United States to 0.6%-1.1% in the Netherlands and Belgium [4].

The spectrum of available management options is broad, spanning multiple medical disciplines, including psychological, hormonal, and surgical interventions. The decision to pursue any or all these interventions varies among individuals, and optimal management is individualized to their specific goals and needs [5]. Some transgender individuals have undergone gender-affirming hormonal therapy (GAHT) as part of their transition process [6-8]. Transgender healthcare is witnessing rapid growth within the medical field, with a rising number of transgender individuals seeking guidance and support for transitioning purposes [9]. Typically, transgender women undergo estrogen treatment to achieve feminization, while transgender men receive androgen therapy to facilitate masculinization, aligning their physical appearance with their gender identity [6,7].

Numerous adverse effects of GAHT have been documented in previous studies, such as deterioration of lipid profiles and cardiovascular events such as myocardial infarction, stroke, and venous thromboembolism [10]. There is a notable gap in the existing knowledge about the impact of GAHT on body composition, insulin resistance, or incidence of diabetes mellitus. Both estrogen and testosterone have demonstrated the ability to modulate insulin sensitivity in cisgender (gender identity as same as assigned sex at birth) women and men [11-15]. However, the precise effect of GAHT on insulin resistance in transgender individuals remains uncertain [5].

There is an imperative need for a comprehensive understanding of the health implications of GAHT for transgender individuals. In this article, we are undertaking a systematic review focusing on investigating the relationship between GAHT and the incidence of type 2 diabetes mellitus and metabolic disturbances including insulin resistance, change in body mass index (BMI), and change in body fat. Our analysis compares transgender individuals undergoing GAHT to men and women in the general population, as well as transgender individuals not receiving GAHT.

## Review

Methods

We used the Preferred Reporting Items for Systematic Reviews and Meta-Analyses (PRISMA) guidelines and principles to design this systematic review and report the results [16].

Inclusion and Exclusion Criteria

We included randomized controlled trials (RCTs), clinical trials, meta-analyses, and observational studies published in English in the past 10 years. Our review includes human studies focusing mainly on transgender individuals relevant to our research question to understand recent relevant research findings in this area comprehensively. Furthermore, we excluded articles on type 1 diabetes, case reports, letters, expert opinions, animal studies, traditional review articles, and unpublished or grey literature.

Search Strategy

We used research literature databases and search engines such as PubMed, PubMed Central (PMC), Google Scholar, and ResearchGate with appropriate keywords and Medical Subject Headings (MeSH) terms to find relevant articles about the topic published in the last 10 years (2014 to 2024). Our search was conducted between December 2023 and February 2024. The date of our last search was February 20, 2024. We combined the main keywords using Boolean "AND" and "OR." We also used the MeSH terms in our PubMed/Medline search. The final combined MeSH terms and keywords used for PubMed, PubMed Central, and Medline are represented in Table 1.

**Table 1:** Search strategy for PubMed GnRH, gonadotropin-releasing hormone; MeSH, Medical Subject Headings; NIDDM, non-insulin-dependent diabetes mellitus

Keywords and MeSH keywords (date: December 2, 2024)	Results
Gender affirming hormonal therapy OR estrogen OR estradiol OR testosterone OR androstenedione OR androgen OR antiestrogen OR antiandrogen OR GNRH analogue OR progestogens OR gonadotropin releasing hormone agonist OR gonadotropin releasing hormone agonist OR pregestational agent OR hormone replacement therapy	532,885
Type 2 diabetes OR insulin resistance OR diabetes mellitus OR non insulin dependent diabetes OR adult onset diabetes OR hyperglycemia OR mature onset diabetes OR maturity onset diabetes mellitus OR NIDDM OR ketoacidosis resistant diabetes OR hyperglycemia	779,603
Transgender individuals OR transsexual OR transsexualism OR Transgenderism OR transexuals OR transgender person OR transgender OR trans men OR trans women OR nonbinary OR genderqueer OR third gender OR cross dressers OR transexual OR transvestite OR trans OR male to female OR female to male OR trans sex OR gender neutral OR intersexual OR bisexual OR homosexual OR gender bending OR ambisexual OR non binary OR trans	6,981,592
(“Gonadal Hormones/adverse effects"[Mesh] OR “Gonadal Hormones/agonists"[Mesh] OR “Gonadal Hormones/deficiency"[Mesh] OR “Gonadal Hormones/metabolism"[Mesh] OR “Gonadal Hormones/pharmacology"[Mesh] OR “Gonadal Hormones/physiology"[Mesh] OR “Gonadal Hormones/standards"[Mesh] OR “Gonadal Hormones/supply and distribution"[Mesh] OR “Gonadal Hormones/therapeutic use"[Mesh])	223,990
("Diabetes Mellitus, Type 2/diagnosis"[Mesh] OR "Diabetes Mellitus, Type 2/drug therapy"[Mesh] OR "Diabetes Mellitus, Type 2/etiology"[Mesh] OR "Diabetes Mellitus, Type 2/metabolism"[Mesh] OR "Diabetes Mellitus, Type 2/physiopathology"[Mesh] OR "Diabetes Mellitus, Type 2/prevention and control"[Mesh] )	148,031
("Transsexualism/complications"[Mesh] OR "Transsexualism/drug therapy"[Mesh] )	415
Combined search strategy of keywords and mesh keywords	3934

Screening

Two writers independently searched the databases for papers that fit the inclusion and exclusion criteria. Where there were disagreements, a third author was included to resolve them. We checked the titles and then the abstracts to select articles that would fit the goal of this study. Twelve articles were selected after the final screening. 

Analysis of Study Quality/Bias

Two authors were involved in the appraisal of the selected articles for quality. They critically evaluated 12 selected studies for quality using standardized quality assessment tools. Eleven studies that qualified as medium or high quality were included in the review. The following tools were used for quality assessment: (1) Newcastle-Ottawa scale for observational studies; (2) Cochrane risk-of-bias assessment tool for RCTs; (3) Assessment of Multiple Systematic Reviews (AMSTAR) tool for systematic reviews and meta-analyses. Quality assessment for observational studies is summarized in Table 2.

**Table 2:** Summary of quality assessment using the Newcastle Ottawa scale for observational studies

Selection	Van Velzen et al. [17]	Auer et al. [18]	Islam et al. [19]	Klaver et al. [20]	Nokoff et al. [21]	Shadid et al. [22]	Suppakitjanusant et al. [23]	Val Valzen et al [24]	Deischinger et al [25]
Representativeness of the exposed cohort	1	1	1	1	1	1	1	1	1
Selection of the non-exposed cohort	1	1	1	1	1	1	1	1	1
Ascertainment of exposure	1	1	1	1	1	1	1	1	1
Demonstration that outcome of interest was not present at the start of the study	1	1	1	1	1	1	1	1	1
Comparability									
Study controls for the most important factor (age)	1	1	1	1	1	1	1	1	1
Study controls for any additional factor(s)	1	1	1	1	1	1	1	1	1
Outcome									
Assessment of outcome	1	1	1	1	1	1	1	1	1
Was follow-up long enough for outcomes to occur?	1	1	1	1	0	1	1	1	0
Adequacy of follow-up of cohorts	1	1	1	1	0	1	1	1	0
Total	9/9	9/9	9/9	9/9	7/9	9/9	9/9	9/9	7/9
Quality	High	high	high	high	medium	high	high	high	high

Quality assessment for RCTs using the Cochrane Risk-of-Bias Tool is summarized in Table 3.

**Table 3:** Comprehensive explanation of how we employed the Cochrane risk-of-bias tool to assess the quality of the randomized controlled trials included in our study. Cochrane Risk-of-Bias Tool (Modified) For Quality Assessment of Randomized Controlled Trials “Yes” in all Domains would place a study at a “low risk of bias.” “No” in any of the domains would place a study at a “high risk of bias.” “Unclear” in any domain would place the study at an “unclear risk of bias.”

Author	Mather et al. [26]
Random sequence generation: Was the allocation sequence already generated?	Yes
Allocation concealment: Was the sequence generation adequately concealed before group assignments?	Yes
Blinding of participants: Was knowledge of the allocated interventions adequately hidden from the participants and personnel after participants were assigned to respective groups?	Yes
Blinding of outcome assessments: Was knowledge of the allocated interventions adequately hidden from the outcome assessors after participants were assigned to respective groups?	Yes
Incomplete outcome data: Were incomplete outcome data adequately addressed?	Yes
Selective reporting: Are reports of the study free of suggestion of selective outcome reporting	Yes
Other sources of bias: Was the study apparently free of other problems that could put it at risk of bias?	Yes
Risk of bias	Low

Quality assessment for Systematic review using the AMSTAR checklist is summarized in Table 4.

**Table 4:** Elaborates on the AMSTAR criteria used to assess the quality of the systematic review we included. AMSTAR, Assessment of Multiple Systematic Reviews; PICO, population, intervention, comparators, outcomes; RCTs, randomized controlled trials; RoB, risk of bias

AMSTAR criteria for Spanos et al. [5]	(Yes, partial yes, no)
1. Did the research questions and inclusion criteria for the review include PICO components?	Yes
2. Did the report of the review contain an explicit statement that the review methods were established prior to the conduct of the review and did the report justify any significant deviations from the protocol?	Yes
3. Did the review authors explain their selection of the study designs for inclusion in the review?	Yes
4. Did the review authors use a comprehensive literature search strategy?	Yes
5. Did the review authors perform study selection in duplicate?	Yes
6. Did the review authors perform data extraction in duplicate?	Yes
7. Did the review authors provide a list of excluded studies and justify the exclusions?	Yes
8. Did the review authors describe the included studies in adequate detail?	Yes
9. Did the review authors use a satisfactory technique for assessing the RoB in individual studies that were included in the review?	No RCT was not included
10. Did the review authors report on the funding sources for the studies included in the review?	Yes
11. If meta-analysis was performed, did the review authors use appropriate methods for statistical combination of results?	No
12. If meta-analysis was performed, did the review authors assess the potential impact of RoB in individual studies on the results of the meta-analysis or other evidence synthesis?	No
13. Did the review authors account for RoB in individual studies when interpreting/discussing the results of the review?	Yes
14. Did the review authors provide a satisfactory explanation for, and discussion of, any heterogeneity observed in the results of the review?	Yes
15. Did the review authors provide a satisfactory explanation for, and discussion of, any heterogeneity observed in the results of the review?	Yes
16. Did the review authors report any potential sources of conflict of interest, including any funding they received for conducting the review?	Yes
Total score	13/16 (high quality)

Data Extraction

After the eligible studies were determined, two investigators examined them for data extraction based on the following: (1) type of study, (2) number of participants, (3) intervention and duration of the study, (4) outcome based on BMI, insulin resistance, and hyperglycemia, and (5) long-term outcomes including incidence of type 2 diabetes.

Results

The search strategy identified 3,934 articles from PubMed, Medline, and PubMed Central, and relevant articles were searched through keywords from Google Scholar (27) and ResearchGate (8), resulting in 35 more articles. After initial screening, 235 articles were selected. Duplicate records (7) were then removed.

**Figure 1:**
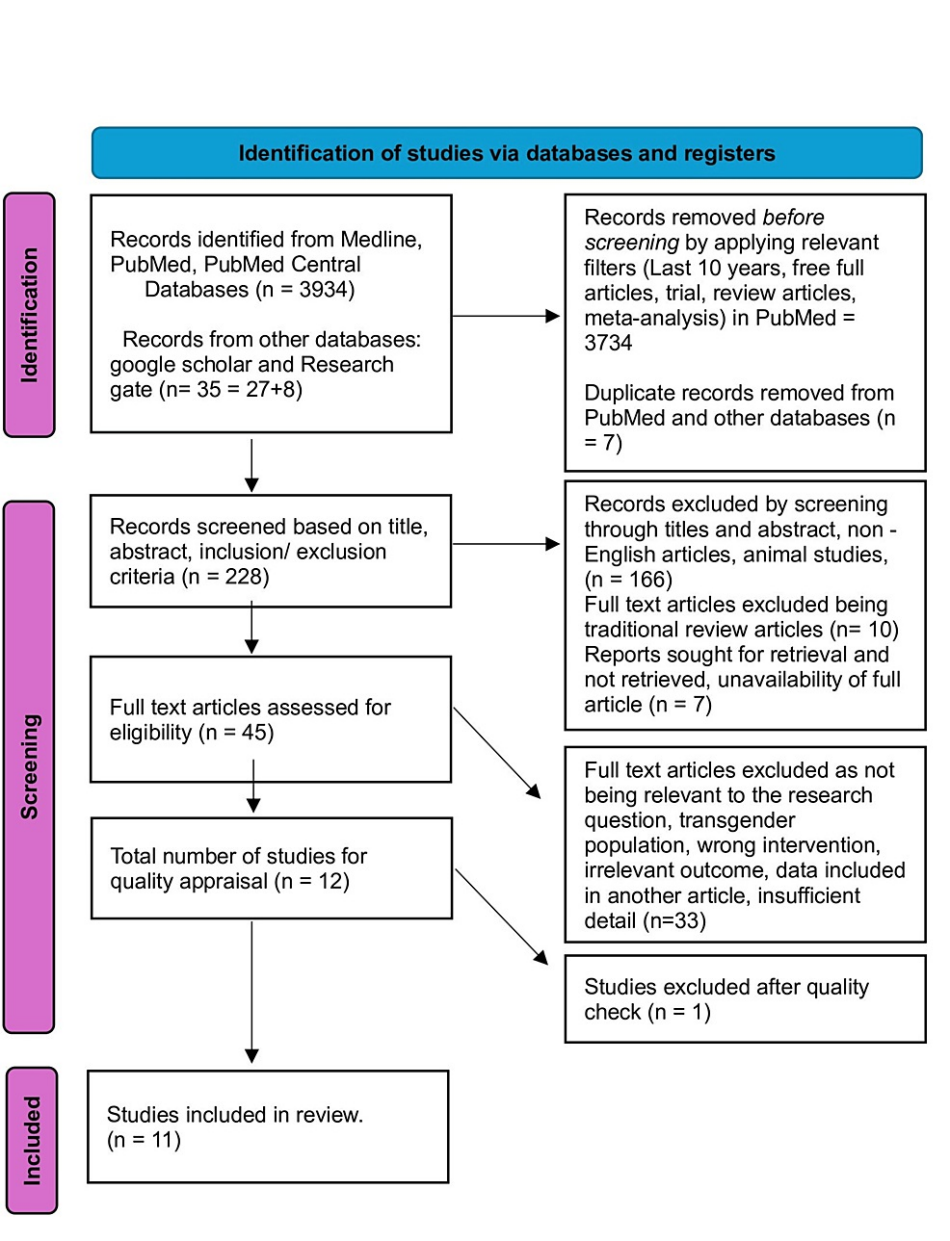
PRISMA 2020 flow diagram n=number Medline, Medical Literature Analysis and Retrieval System Online; PRISMA, Preferred Reporting Items for Systemic Reviews and Meta-Analyses

Two individual investigators then screened the remaining articles (n=228) based on titles, abstracts, full text, and detailed inclusion-exclusion criteria. After the meticulous screening, we were left with 12 articles pertaining to our research question. These 12 studies were included for a thorough quality/bias assessment using standardized quality assessment tools. One study was excluded after quality appraisal, and the final 11 studies were included in this systematic review. The PRISMA 2020 flow diagram is depicted in Figure 1.

The final review comprised 11 selected studies, including seven cohort studies, two cross-sectional studies, one secondary analysis of RCT, and one systematic review. Five articles compared insulin resistance and sensitivity among transgender women and transgender men undergoing GAHT with controls. Three articles compared body fat and BMI differences among those who underwent GAHT compared to the controls. Two studies compared diabetes incidence among those who underwent GAHT. One study researched the role of sex hormonal supplementation in men and pre- or post-menopausal women, as well as its link to diabetes incidence. The characteristics and results of the 11 research articles in our systematic review are shown in Table 5.

**Table 5:** Summary of study characteristics AFAB, assigned female at birth; AMAB, assigned male at birth; BMI, body mass index; CI, confidence interval; DPP, Diabetes Prevention Program; EHR, electronic health record; GAHT, gender-affirming hormone therapy; GNRHa, gonadotropin-releasing hormone agonist; HbA1c, hemoglobin A1C; HOMA-IR, Homeostatic Model of Insulin Resistance; HR, hazard ratio; HT, hormonal therapy; insulin sensitivity, 1/fasting insulin; OGTT, oral glucose tolerance test; SAT/VAT, subcutaneous adipose tissue/visceral adipose tissue; SHBG, sex hormone binding globulin; SIR, standardized incidence ratio; T2DM, type 2 diabetes mellitus

Author and year of publication	Intervention/time of study	Number of patients	Type of study/duration of study	Outcome measures, main findings, result/conclusion
Spanos et al. (2020) [5]	GAHT receiving with testosterone or estradiol	26 studies included	A systematic review of 26 studies: 2 cross-sectional, 21 prospective uncontrolled, and 3 prospective controlled studies	TW decreases lean mass, increases fat mass, and may worsen insulin resistance. TM gained lean mass and lost fat mass.
Van Velzen et al. (2022) [17]	In transgender women, hormone therapy consists of anti-androgens in combination with estrogens. In transgender men, hormone therapy consists of testosterone.	2,585 transgender women and 1,514 transgender men	Cohort study between 1972 and 2018	Transgender women (N = 2,585, 90 cases; SIR 0.94; 95% CI 0.76-1.14) and transgender men (N = 1,514, 32 cases; SIR 1.40; 95% CI 0.96-1.92) did not vary in the incidence of T2DM.
Auer et al. (2018) [18]	GAHT with 1000 mg testosterone for transgender men; cyproterone acetate 50 mg and 2 mg estradiol valerate/transdermal patch dependent on age	24 transgender women and 45 transgender men	Prospective cohort study; baseline and 12 months of HT	Transgender women had worsened insulin resistance, HOMA-IR, (mean 1.7, 95% CI 1.3–2.1, P = 0.001), and increased early insulin response. Transgender men had improvement in hepatic insulin sensitivity, HOMA-IR decreased, mean 2.4, 95% CI 1.8–3.0, P = 0.004).
Islam et al. (2022) [19]	GAHT received as per HER linkages to prescription data using national drug codes	2,869 transfeminine members matched to 28,300 cisgender women and 28,258 cisgender men; 2,133 transmasculine members matched to 20,997 cisgender women and 20,964 cisgender men	EHR- based cohort study 9 years from 2006 through 2014, with follow-up through 2016	Prevalence and incidence of T2DM more in the transfeminine cohort compared to cisgender females, with an odds ratio of 1.3 and 95 % CI 1.1-1.5, and no significant difference compared to cisgender males.
Klaver et al. (2022) [20]	GAHT: cyproterone acetate with oral estradiol or a transdermal estradiol patch in transgender women. Transgender men: testosterone formulations	179 transgender women, and 162 transgender men	Prospective observational study before and after one year of hormone therapy in trans persons	Transgender women: total body fat increased by 4 kg (95% CI 3.4-4.7), with no changes in visceral fat. Transgender men: body fat decreased by 2.8 kg (95% CI 2.2-3.5), with no changes in visceral fat
Nokoff et al. (2021) [21]	Therapy with an agonist of GnRHa	Transgender males (n=9) on GnRHa were compared with cisgender females (n=14). Transgender females (n=8) on GnRHa were compared with cisgender males (n=17, age: 12.5–15.5)	Cross-sectional study	Transgender youth on a GnRHa have lower estimated insulin sensitivity and higher glycemic markers and body fat than cisgender controls with similar characteristics. Transgender females (p=0.028, p=0.035) and transgender males (p=0.031, p=0.01) had lower 1/fasting insulin and higher HOMA-IR, respectively
Shadid et al. (2020) [22]	GAHT with 1,000 mg testosterone for transgender men; cyproterone acetate 50 mg, and 2 mg estradiol valerate/transdermal patch dependent on age.	35 transgender males and 55 transgender females	Prospective cohort study at baseline and 12 months of HT	HOMA-IR (2.2 ± 0.3 vs. 1.8 ± 0.2; P = 0.06) and fasting insulin (-1.4 ± 0.8 mU/L; P = 0.08) tended to decline in transgender men. HOMA-IR (1.7 ± 0.1 vs. 2.4 ± 0.2; P < 0.01) and insulin (3.4 ± 0.8 mU/L; P < 0.01) tended to rise in transgender women. The tendency is for post-OGTT incretin responses and insulin sensitivity to rise with masculinization and fall with feminization.
Suppakitjanusant et al. (2020) [23]	GAHT: transgender women: oral, transdermal or intramuscular estradiol along with spironolactone. Transgender men: intramuscular or transdermal testosterone.	227 subjects	Retrospective cohort study between 2000 and 2018	Following the commencement of GAHT in a single U.S. center, BMI of transgender women increased significantly (p-value 0.004) but not that of transgender men. BMI seems to be stable in transgender women and transgender men after three to six years of GAHT.
Van Velzen et al. (2019) [24]	Transdermal or intramuscular application of testosterone and oral or transdermal delivery of estrogen (plus cyproterone)	242 transgender women and 188 transgender men	Prospective cohort study before and after 12 months of HT from 2010 to 2017.	BMI slightly increased in transgender men, 23.5 (21.3–28.2), and transgender women, 22.8 (20.5–26.5), p < 0.05, but did not vary between administration routes. Transgender men showed unfavorable changes in their lipid profiles, while transgender women showed beneficial changes.
Deischinger et al. (2022) [25]	GAHT	38 transgender persons (16 AFAB, 22 AMAB) and 33 cisgender persons (16 cisgender females, 17 cisgender males)	Cross-sectional mono-center pilot study between 2013 and 2020	HbA1c (p=0.031) was significantly lower in AMAB than cisgender males; fasting insulin (p=0.034) and HOMA-IR (p=0.037) were higher in AMAB comparison to cisgender males and cisgender females (HOMA-IR: p = 0.014, fasting glucose: p < 0.001); SAT/VAT-ratio (p = 0.013) was higher in AMAB than in cisgender males, consistent with expected changes under estrogen GAHT.
Mather et al. (2015) [26]	Randomized to receive intensive lifestyle intervention, metformin, or placebo	With regard to the 2,898 DPP participants, there were 969 men, 948 premenopausal women who did not use exogenous sex hormones, 550 postmenopausal women who did not take exogenous sex hormones, and 431 postmenopausal women who did take exogenous sex hormones.	Multicenter randomized clinical trial, secondary analysis including 27 academic institutions in the United States	Estrogens and testosterone predicted diabetes risk in men but not in women. Diabetes risk was directly associated with estrone (HR, 1.101; 95% CI, 1.059–1.145), estrone sulfate (HR, 1.003; 95% CI, 1.001–1.005), and estradiol (HR, 1.130 95 % CI, 10.45–1.221). SHBG and its polymorphisms did not predict risk in men or women. Diabetes risk is more potently determined by obesity and glycemia than by sex hormones.

Discussion

Several studies that attempt to improve our knowledge of the relationship between GAHT and insulin resistance are compiled in this systematic review. This study also aims to clarify the implications of GAHT on BMI, body fat, gender disparities, and the occurrence of type 2 Diabetes. We investigated the results and insights gained from an in-depth analysis of these studies, offering significant novel perspectives on how GAHT affects insulin resistance and diabetes. We explored specific components of this relationship in the sections that follow to gain a deeper understanding of how GAHT affects the metabolic health of the transgender population. This study will give more insight into the importance of monitoring the metabolic status of transgenders undergoing GAHT.

Insulin Resistance and GAHT

Insulin resistance has been established to lead to type 2 diabetes mellitus. Its correlation has already been found in the medical literature [27]. Therefore, we focused on studies in which insulin sensitivity or resistance was monitored and measured in transgender patients undergoing hormonal therapy.

Auer et al. conducted a cohort study with 24 transgender women and 45 transgender men to study metabolic syndrome in transgender individuals undergoing GAHT. They concluded that transgender women's insulin resistance and risk of diabetes are exacerbated by GAHT. In contrast, transgender men have improved insulin sensitivity after undergoing GAHT. This was a cohort cross-over study of 12 months [18]. This study, however, comprised patients treated in a single hospital, Ghent University Hospital in Belgium, and the sample size was smaller, with a study duration of one year.

Similarly, Shadid et al. conducted a cross-over cohort study for a year in 35 transgender men and 55 transgender women. The data were from the ENIGI (European Network for the Investigation of Gender Incongruence) study, which concluded that insulin sensitivity increases within transgender men who underwent masculinizing hormonal therapy with testosterone, but insulin sensitivity decreases in a transgender female who underwent feminizing hormonal treatment with estrogen. They also concluded that this finding correlates with the fact that hormonal changes bring out changes in lean mass and fat mass; testosterone decreases fat mass, and estrogen increases fat mass. These body composition changes could have led to insulin resistance [22]. This study, however, could not determine if men or women differ in insulin sensitivity based on their sex on an intrinsic level. This study also did not find any changes in the glucose levels.

Interestingly, a systematic review of 26 trials conducted in 2020 by Spanos et al. concluded that feminizing hormone therapy (estradiol, with or without anti-androgen drugs) in transgender women reduces lean mass, increases fat mass, and increases insulin resistance. It showed that insulin resistance in transgender women worsens with GAHT [5]. However, it is not entirely conclusive due to the scarcity of RCTs, and the review mainly contained observational studies with small cohorts and short follow-up periods. Further research with larger sample sizes and RCTs would be beneficial to corroborate these findings and enhance our understanding of the relationship between insulin resistance and GAHT.

In contrast to the findings, Nokoff et al. conducted a study in which transgender men and transgender women taking gonadotropin-releasing hormone agonist have higher insulin resistance. Both transgender men and transgender women had lower insulin sensitivity and higher percent body fat than cisgender women and cisgender men whose age, sex, and BMI matched, respectively [21]. However, this was a cross-sectional study with a smaller sample size. Nine transgender men were compared to 14 cisgender women and eight transgender women to 17 cisgender men. Thus, the data would not be very generalizable, as longitudinal studies with larger sample sizes would be more meaningful.

Furthermore, Deischinger et al. did a study with 38 transgender and 33 cisgender controls. They found fasting insulin and HOMA-IR (Homeostatic Model of Insulin Resistance) to be significantly higher in transgender women compared to both cisgender sexes [25]. This study had weight- and age-matched individuals to correct for weight or body composition-related bias. In contrast to these changes, hemoglobin A1C (HbA1c), the indicator for diabetes, was lower in transgender women. However, the study's conclusions might be less broadly applicable because of the cross-sectional methodology and limited sample size.

Nevertheless, monitoring insulin sensitivity parameters regularly in transgender people undergoing hormonal therapy is extremely important, as indicated by all studies. Overall, these studies provide valuable and insightful information regarding the connection between GAHT and insulin resistance. However, further research is needed to fully understand the complex relationship and the potential influence of insulin sensitivity in transgender population undergoing hormonal therapy. These results add to the body of knowledge already in existence and highlight the significance of thorough research to improve our understanding of the interaction between insulin resistance and GAHT in the setting of transgender patients.

GAHT and Diabetes Incidence

In 2021, CDC (Centers for Disease Control and Prevention) data show that 38.4 million people, or 11.6% of the total U.S. population, had diabetes [28]. This includes 25% gay or bisexual men and 14% lesbian or bisexual women [11]. These data suggest that there is a higher risk for type 2 diabetes in transgender population compared to the general population.

Researchers from the Amsterdam Cohort of Gender Dysphoria looked at the possibility of a link between the risk of diabetes and receiving GAHT. They included transgender patients getting GAHT who made at least one clinic follow-up visit. The National Civil Record Registry data were used as a reference group to determine the prevalence of type 2 diabetes among the Dutch population. The transgender population comprised of 2,585 transgender women and 1,514 transgender males. The median follow-up period was 11.3 years in transgender women and 5.2 years in transgender men. During the study period, 99 transgender women and 32 transgender men developed diabetes; the median age at diagnosis was 50 and 55 years, respectively [17]. Transgender men and women did not have a higher standardized incidence ratio for diabetes than the control group of the whole Dutch population.

Similarly, a study conducted by Islam et al. found no differences in the baseline prevalence or incidence of diabetes between transgender women and reference cisgender male and female controls following the initiation of GAHT. A person had to meet two or more criteria to be diagnosed with diabetes: hemoglobin A1c ≥ 6.5%, fasting glucose ≥ 126 mg/dL, or random glucose values ≥ 200 mg/dL. They also needed to be taking medication for their diabetes [19]. The study, however, noted that type 2 diabetes cases were higher in the transgender female group (hazard ratio, 1.4; 95% CI, 1.1-1.8) compared to cisgender females but not compared to cisgender males. This was regarded as a well-known gender disparity in the general population, with males being more at risk of type 2 diabetes than females. Thus, they concluded that the risk of diabetes is due to gender differences rather than GAHT.

In contrast with the findings in the above studies, Mather et al. conducted a study that aimed to correlate the impact of testosterone and estrogen hormonal supplementation with the risk of diabetes and blood glucose. This study concluded that diabetes risk is determined predominantly by obesity rather than sex hormones. Nonetheless, the study among men had shown that lower testosterone and higher estrogen were associated with an increased risk of diabetes. However, no difference was seen with hormonal therapy on the diabetes risk in women [26]. However, it is essential to note that the population in this study did not comprise transgender individuals but men, pre-menopausal, and post-menopausal women getting sex hormonal supplementation.

While it is conceivable that neither insulin resistance nor increased BMI has reached levels indicative of type 2 diabetes mellitus, they may pose a potential risk in the future, mainly if observed over an extended duration. Nevertheless, there are no controlled studies that could be included about the transgender population to look at the incidence or prevalence of diabetes.

Effects of GAHT on BMI and Body Fat

The influence of BMI and body fat resulting in type 2 diabetes mellitus has been extensively investigated. Being overweight or obese is known to be a significant risk factor for diabetes and is brought on by a higher BMI [27]. In this section, we are exploring the potential side effects of GAHT and the possible role of GAHT in modulating BMI and body fat.

Klaver et al. conducted a crossover cohort study of one year with 179 transgender women and 162 transgender men. Transgender women's body fat increased, and transgender men's total body fat decreased. Still, visceral fat accumulation was unrelated to any unwanted cardiometabolic adverse effects due to GAHT. The study emphasized the relation between visceral fat and insulin resistance, concluding that changes in visceral fat were unrelated to GAHT for one year. The study observed significant changes in insulin resistance. It was only associated with an increase in total body fat but not with visceral fat, especially in transgender women [20]. This study was conducted in a large cohort of transgender individuals and showed a difference in body fat distribution but no significant difference in insulin resistance.

Suppakjitjanusant et al. conducted a retrospective cohort study with 227 individuals. Despite the initial increase in BMI in transgender women after starting GAHT within the first 24 months, BMI was stable within the next few years [23]. This study had a more extended observational period than other studies and did not link GAHT to BMI increment.

In contrast to the previous studies, Van Valzen et al. executed a prospective, observational study for one year with 242 transgender women and 188 transgender men. BMI slightly increased in both transgender men and transgender women [24]. Insulin resistance or incidence of diabetes was not reported in this study as well.

Furthermore, more extensive studies with more extended observational periods or RCTs appear necessary to answer the question of the impact of GAHT on insulin resistance, BMI, and incidence of diabetes mellitus. Until then, our findings support recommendations to monitor transgender persons both before GAHT and undergoing GAHT concerning insulin resistance and the development of prediabetes or DM.

Strengths and uniqueness of the study

This systematic review is unique because of several of its vital attributes. The thorough search approach covered several databases, reducing the possibility of missing pertinent research. The results are more universally applicable since many studies from different countries and patient demographics are included. Strict inclusion and exclusion criteria ensure the selection of research that meets rigorous methodological requirements. This study offers a current assessment of the state-of-the-art knowledge about the incidence of type 2 diabetes and metabolic changes among transgender community following GAHT by integrating the existing evidence.

Limitations of the study

There are certain restrictions on this systematic review. Firstly, this study only included papers that were published in English. We limited our analysis to RCTs, systematic reviews, and observational studies. This approach may have caused us to overlook essential studies in other languages or types, which could have enhanced the strength of the review. Also, the studies that did not include the transgender community were excluded, except for one study related to our research question. Finally, there were some publications that fulfilled our inclusion criteria but were not freely accessible and were not included in this analysis.

## Conclusions

Our study showed that GAHT increased BMI and insulin resistance in transgender population compared to their cisgender counterparts and transgender population not on GAHT. More transgender women had increased insulin resistance and increased BMI compared to transgender men in most of the studies. However, our study did not show any increase in the incidence of diabetes among transgender individuals receiving GAHT. Clinicians can be recommended to monitor metabolic risk by regularly checking blood glucose, BMI, and HBA1c in their GAHT-treated patients. Given the limited research available, larger-scale clinical trials are needed to fully understand the long-term impact of GAHT on insulin resistance and the incidence of type 2 diabetes mellitus in transgender patients.
